# Potential Antimicrobial Activity of Galloyl-Flavonoid Glycosides From *Woodfordia uniflora* Against Methicillin-Resistant *Staphylococcus aureus*

**DOI:** 10.3389/fmicb.2021.784504

**Published:** 2021-11-26

**Authors:** Jae Sik Yu, Ji-Hoon Kim, Luay Rashan, Inseo Kim, Wonsik Lee, Ki Hyun Kim

**Affiliations:** ^1^School of Pharmacy, Sungkyunkwan University, Suwon, South Korea; ^2^Biodiversity Unit, Research Center, Dhofar University, Salalah, Oman

**Keywords:** *Woodfordia uniflora*, flavonoid, antimicrobial activity, biofilm formation, methicillin-resistant *Staphylococcus aureus*

## Abstract

Antibiotic-resistant infections are a growing problem; to combat multi-drug resistant bacterial infections, antibiotics with novel mechanisms of action are needed. Identification of potent bioactive natural products is an attractive avenue for developing novel therapeutic strategies against bacterial infections. As part of our ongoing research to explore bioactive natural products from diverse resources, we investigated the antimicrobial compounds from *Woodfordia uniflora*, a flowering shrub unique to the Dhofar region of Oman. The plant has been used as a remedy for skin infections in Oman. However, to date, no study has examined the antimicrobial compounds in *W. uniflora*. Phytochemical analysis of the methanolic extract of *W. uniflora* leaves in combination with LC/MS-based analysis allowed us to isolate and identify four flavonoid-type analogs (**1–4**), procyanidin B3-3-*O*-gallate (**1**), rhamnetin 3-*O*-(6″-galloyl)-β-D-glucopyranoside (**2**), rhamnetin 3-*O*-α-L-rhamnopyranoside (**3**), and quercetin 3-*O*-(6″-galloyl)-β-D-glucopyranoside (**4**). The isolates have a novel mechanism of action; the compounds inhibit biofilm formation in methicillin-resistant *Staphylococcus aureus* (MRSA) and synergize with methicillin. Our metabolite analysis revealed that this synergizing activity by compounds was achieved by remodeling metabolism including central carbon metabolism and glutamine biosynthesis that resulted in abnormal cell formation and reduction in biofilm formation of MRSA. Taken together, these findings provide experimental evidence that rhamnetin 3-*O*-(6″-galloyl)-β-D-glucopyranoside (**2**) and quercetin 3-*O*-(6″-galloyl)-β-D-glucopyranoside (**4**) can be considered as potential therapeutic agents for the treatment of methicillin-resistant *S. aureus*-associated diseases.

## Introduction

Antibiotic-resistant bacterial infection remains a major threat to public health; the surge of hospitalized patients with COVID-19 increases the probability of transmission of nosocomial bacterial infections between the patients. Antibiotic-resistant *Staphylococcus aureus* is a leading cause of death associated with antibiotic resistance globally (World Health Organization, 2017). To treat *S. aureus* infections, β-lactams have been primarily used; however, most *S. aureus* infections were found to be methicillin-resistant (methicillin-resistant *S. aureus*: MRSA). Currently, other antibiotics such as vancomycin and daptomycin, having different mechanisms of action, have been used to treat MRSA infections; however, the number of clinical failures due to antibiotic resistance is increasing (Spagnolo et al., 2014; Stefani et al., 2015). Therefore, there is an urgent need to develop novel antibiotics and make the existing ones more effective.

As part of ongoing research to discover natural bioactive compounds (Ha et al., 2020; Lee S. et al., 2020; Lee S. R. et al., 2020; Yu et al., 2020a), we investigated the bioactive compounds from *Woodfordia uniflora* collected in Oman. *W. uniflora* is a tall slender shrub belonging to Lythraceae and a native plant species of Oman. This plant is abundant throughout the Dhofar region of Oman and in the high-altitude regions of East Africa and the Middle East. *Woodfordia* species have been used in Asia for medicinal purposes by different ethnic groups (Graham, 1995). In southern Asia, dried flowers have been used as an astringent for treating ulcers, wounds, and diarrhea; its leaves have been used for their antibiotic and sedative properties (Dhar et al., 1968; Das et al., 2007; Kaur and Kaur, 2010; Nitha et al., 2012). The flowers of *Woodfordia* species have also been used as traditional ayurvedic fermented drugs in India (Kroes et al., 1993). Previous studies of *Woodfordia* species have shown an abundance of tannins and phenolics, but most of these studies were based on *W. fruticosa* (Nitha et al., 2012), which is morphologically similar to *W. uniflora*. In general, *W. uniflora* has been used locally in the Dhofar region, Oman, to treat skin infections and wounds. However, to the best of our knowledge, no study has been conducted on phytochemicals in *W. uniflora*, and no scientific investigation has proven its ethnopharmacological activity. Hence, we investigated the bioactive phytochemicals from *W. uniflora* (Yu et al., 2020b). In our recent study on the phytochemicals of *W. uniflora* leaves—the first phytochemical examination of the species—we identified 19 polyphenols including three new polyphenols, using antifungal bioactivity-driven LC/MS-based analysis; strong antifungal compounds against *Cryptococcus neoformans* and *Candida albicans* human fungal pathogens (Yu et al., 2020b) were found. In particular, the new polyphenol (±)-woodfordiamycin showed antifungal activity against *C. neoformans* at the lowest minimum inhibitory concentration of 0.78 μg/mL, ∼10-fold lower than that of fluconazole positive control (Yu et al., 2020b). In addition to antifungal compounds, an anti-inflammatory polyphenol, catechin-7,4-*O*-digallate was also identified in our recent study (Kim et al., 2021). Its anti-inflammatory effects were found to be mediated via the inhibition of nuclear factor kappa-light-chain-enhancer of activated B cells (NF-κB) activation and regulation of the expression of pro-inflammatory cytokines such as IL-6 and IL-1β (Kim et al., 2021).

The present study continues the examination of phytochemicals found in *W. uniflora* by exploring the antimicrobial compounds from the methanolic extract of *W. uniflora* leaves in the combination with liquid chromatography/mass spectrometry (LC/MS)-based analysis. The phytochemical investigation led to the isolation and identification of four flavonoid-type analogs (**1–4**). Further investigation of MRSA revealed that compounds **1–4** effectively inhibited biofilm formation independent *of agrA* induction, and compounds **2** and **4** showed a strong synergistic effect with methicillin. In our fluorescence microscopy and metabolite analysis, we found that treatment with compounds **2** or **4** led to metabolic remodeling resulting in a septal defect and abnormal cell size that contributed to reduced biofilm formation in MRSA. Herein, we describe the isolation and structural characterization of compounds **1–4** as well as their antimicrobial potency against methicillin-resistant *S. aureus*.

## Materials and Methods

### General Experimental Procedures

Optical rotations were measured using a Jasco P-2000 polarimeter (Jasco, Easton, MD, United States). Electronic circular dichroism (ECD) spectra were measured using a Jasco J-1500 spectropolarimeter (Jasco). Ultraviolet (UV) spectra were acquired on an Agilent 8453 UV-visible spectrophotometer (Agilent Technologies, Santa Clara, CA, United States). NMR spectra were recorded using a Bruker AVANCE III HD 850 NMR spectrometer with a 5 mm TCI CryoProbe, operated at 850 MHz (^1^H) and 212.5 MHz (^13^C). Preparative HPLC was performed using a Waters 1525 Binary HPLC pump with a Waters 996 photodiode array detector (Waters Corporation, Milford, MA, United States) and an Agilent Eclipse C_18_ column (250 × 21.2 mm, 5 μm; flow rate: 5 mL/min; Agilent Technologies). Semi-preparative HPLC was performed on a Shimadzu Prominence HPLC System with SPD-20A/20AV Series Prominence HPLC UV-Vis detector (Shimadzu, Tokyo, Japan) and a Phenomenex Luna C_18_ column (250 × 10 mm, 5 μm; flow rate: 2 mL/min; Phenomenex, Torrance, CA, United States). LC/MS analysis was performed on an Agilent 1200 Series HPLC system equipped with a diode array detector and 6130 Series ESI mass spectrometer, and an analytical Kinetex C_18_ 100 Å column (100 × 2.1 mm, 5 μm; flow rate: 0.3 mL/min; Phenomenex). Silica gel 60 (230–400 mesh; Merck, Darmstadt, Germany) and RP-C_18_ silica gel (Merck, 230–400 mesh) were used for column chromatography. Sephadex LH-20 (Pharmacia, Uppsala, Sweden) was used as the packing material for molecular sieve column chromatography. Thin-layer chromatography was performed using precoated silica gel F_254_ plates and RP-C_18_ F_254s_ plates (Merck), and spots were detected under UV light or by heating after spraying with anisaldehyde-sulfuric acid.

### Plant Material

*W. uniflora* leaves were collected from the valleys of Ain Jarziz, approximately 17–18 km east of Salalah, Dhofar (Oman), during June–August 2017. The leaves were dried at room temperature, macerated into a fine powder, and stored at room temperature. The plant was classified by Alan Radcliffe-Smith (Royal Botanic Gardens, Kew, England) (Miller and Morris, 1988). A voucher specimen of the material (W-2017) has been deposited at the Biology Department, Sultan Qaboos University, Sultanate of Oman.

### Extraction and Isolation

Finely ground *W. uniflora* leaves (100 g) were extracted with 90% MeOH (500 mL) and stirred at room temperature for 12 h. The extracts were collected, filtered through Whatman filter paper No. 1, and the filtrates were concentrated under vacuum using a rotary evaporator. The resultant crude extract (9.4 g) was suspended in distilled water (700 mL) and subjected to solvent partitioning with EtOAc and *n*-BuOH, yielding the EtOAc-soluble (7.1 g) and *n*-BuOH-soluble (0.7 g) fractions. Both fractions were analyzed by LC/MS using a comparison with our in-house UV library, confirming that the EtOAc-soluble fraction had major peaks with characteristic UV patterns of flavonoids and relatively high molecular ion signals at *m/z* 600–750. The EtOAc-soluble fraction (7.0 g) was further fractionated using a Diaion HP-20 column using a gradient solvent system of MeOH (100% H_2_O-50% MeOH-100% MeOH) to obtain three fractions, W1 (1.8 g) from 100% H_2_O elution, W2 (2.2 g) from 50% MeOH elution, and W3 (3.1 g) from 100% MeOH elution. LC/MS analysis of the three fractions (W1–W3) showed that the major peaks detected in the EtOAc-soluble fraction were mainly present in the W2 and W3 fractions. The W2 fraction (2.2 g) was further subjected to Sephadex LH-20 column chromatography with 100% MeOH elution to obtain four subfractions (W21–W24). Based on the monitoring by LC/MS-based analysis of the subfractions, the W23 subfraction (840 mg) was separated by preparative reversed-phase HPLC with MeOH/H_2_O (20–80% gradient system) to produce six subfractions (W231–W236), and compound **1** (*t*_R_ 25.1 min, 20.7 mg) was isolated from the W232 subfraction (121 mg) by semi-preparative HPLC with an isocratic system of 23% MeOH/H_2_O. In addition, the W3 fraction (3.1 g) was subjected to Sephadex LH-20 column chromatography using 100% MeOH to obtain four subfractions (W31–W34). The W33 subfraction (280 mg) was further fractionated by preparative reversed-phase HPLC with MeOH/H_2_O (10–80% gradient system) to obtain six subfractions (W331–W336). The W335 subfraction (84 mg) was purified by semi-preparative HPLC with an isocratic system of 50% MeOH/H_2_O to yield compounds **2** (*t*_R_ 32.5 min, 2.5 mg) and **4** (*t*_R_ 13.0 min, 1.2 mg). Finally, the W336 subfraction (35 mg) was separated by semi-preparative HPLC with an isocratic system of 70% MeOH/H_2_O to yield compound **3** (*t*_R_ 25.0 min, 1.3 mg).

### Bacterial Strains and Growth Conditions

The MRSA strain *S. aureus* USA300 (TCH1516) was used in this study. *S. aureus* strains were maintained aerobically in tryptic soy broth (TSB, BD) or TSB 1.5% agar at 30°C with shaking at 250 rpm. Methicillin was purchased from Thermo Scientific and used as denoted. Mueller Hinton Broth (MHB, BD) was used for antibiotic susceptibility testing.

### *S. aureus* Biofilm Formation Assay

The biofilm assay was performed as described (Bauer et al., 2013). Briefly, *S. aureus* (strain USA300) was grown in flat-bottomed 96-well polystyrene tissue culture-treated microtiter plates (Thermo) in 2 mL of TSB overnight (16–18 h) at 37°C. Then, the bacterial culture was inoculated in 200 μL/well TSB supplemented with 1% glucose to induce biofilm observed at OD_600_ = 0.01 in a 96-well plate. Compounds were added to the assay plate at concentrations of 5, 20, and 50 μg/mL in triplicate. An equal amount of DMSO was added to untreated cells. The assay plates were then incubated at 37°C for 24 h without shaking. After 24h incubation, the plates were washed twice with 1X phosphate-buffered saline (PBS) to remove non-adherent bacteria and fixed with 99% (v/v) methanol for 15 min at RT. The plates were dried and stained with 2% crystal violet (Sigma-Aldrich) for 15 min at room temperature (RT) (Merritt et al., 2005). After removing residual CV from each well by washing with PBS, the biofilm stained with CV was recovered in 95% (v/v) ethanol for 30 min at RT. The stained CV eluted from the cells was measured at 570 nm using a microplate reader (BioTek, Synergy HTX).

### Quantification of *agrA* Expression

Bacterial cultures were inoculated into 2 mL of TSB containing 5 μg/mL to an OD of 0.01. After incubation for 24 h at 30°C, total RNA was extracted from each strain using the RNeasy Protect Bacteria Mini Kit (QIAGEN). cDNA was synthesized from the isolated RNA using RNA to cDNA EcoDry Premix (Takara). We designed the sequences of the primers as follows: *agrA*, sense primer; CCTCGCAACTGATAATCCTTATG, antisense primer; ACGAATTTCACTGCCTAATTTGA, *gadph*: sense primer, TGCAAGGTCGTTTCACAGGT, antisense primer; GGGATGATGTTTTCTGCCGC. Cycling conditions were as follows: 95°C for 15 s, 40 cycles of 95°C for 15 s, 55°C for 30 s, and 72°C for 30 s; followed by melting curve analysis at 95°C for 15 s, 60°C for 1 min, and 95°C for 15 s.

### Checkerboard Assay

A checkerboard assay was performed in a 96-well plate to examine the antibacterial synergy effect of methicillin (Thermo) and the identified compounds as described (Rajagopal et al., 2016). An overnight culture to an OD_600_ of 0.1 in TSB was reinoculated at 30°C to reach an OD of 1.0 for the assay. Following a twofold serial dilution of antibiotics and compounds in 150 μL of MHB media, the culture was inoculated to an OD_600_ of 0.002. After incubation at 30°C for 16 h with shaking, bacterial growth was measured at OD_600_ (BioTek Synergy HTX).

### Fluorescence Microscopy

To examine the potential effects of the identified compounds on the bacterial cell envelope (the bacterial cell wall and membrane), fluorescence microscopy was used. USA300/pLow-ftsZ-GFP was incubated overnight in TSB supplemented with 5 μg/mL erythromycin (Liew et al., 2011). The culture was diluted at an OD of 0.05 and when OD = 0.5, bacterial cells were treated with 5 or 20 μg/mL compound and incubated at 37°C for 1 h. After the cell membrane was stained using FM™ 4-64 Dye (Invitrogen) for 10 min, the cells were pelleted, washed twice in PBS, and mounted on microscope slides with agarose pads. The cells were imaged using a microscope system (Leica DM6 FS) and analyzed using LAS X software (Leica).

### Metabolite Extraction and Liquid Chromatography-Time-of-Flight Mass Spectrometry

Metabolite extraction and analysis were performed as previously described (Eoh and Rhee, 2014). USA300 OD_600_ was incubated with the compounds at 30°C for 1 h. Then, USA300 was metabolically quenched by a mixture of methanol/acetonitrile/H_2_O (40:40:20), and metabolites were extracted by mechanical lysis with 0.09–0.15 mm glass beads using a tissue homogenizer (Precellys, Bertin Technologies). Lysates were filtered across a 0.2-μm microcentrifuge filter, and total protein was determined using a BCA Protein Assay kit (Thermo Scientific). Extracted metabolites were separated using a 3 × 150 mm, 2.7 μm Poroshell 120 HILIC column (Agilent Technologies). The mobile phase consisted of solvent A (ddH_2_O with 0.2% formic acid) and solvent B (acetonitrile with 0.2% formic acid). The gradient used was as follows: 0–2 min, 85% B; 3–5 min, 80% B; 6–7 min, 75%; 8–9 min, 70% B; 10–11.1 min, 50% B; 11.1–14 min 20% B; 14.1–24 min 5% B followed by a 10 min re-equilibration period at 85% B at a flow rate of 0.4 mL/min. An Agilent Mass 6230 time-of-flight (TOF) coupled to an Agilent 1,290 liquid chromatography (LC) system was used to identify metabolites. The detected ions were considered metabolites based on mass-retention time identifiers for masses showing the expected distribution of accompanying isotopomers. Metabolites were identified using the Agilent MassHunter Profinder software with a mass tolerance of ± 0.005 Da.

### Cell Cytotoxicity Assay

Cell cytotoxic effects of compounds **2** and **4** were measured using CytoTox-Glo™ assay (Promega, Madison, WI, United States) (Kesanakurti et al., 2012). Briefly, A549 cells were seeded in a white bottom 96-well plates at a density of 5 × 10^4^ cells per well. After overnight incubation, cells were treated with a series of compound concentrations (0–20 μg/mL diluted by two-fold) for 16 h, followed by the treatment with a substrate that luminesces upon reaction with intracellular organelle but cannot cross the intact membrane of live cells. The fluorescence intensity was measured using a Synergy HTX multi-mode plate reader (BioTek).

## Results and Discussion

### Isolation and Identification of Compounds **1–4** From *W. uniflora*

**FIGURE 1:**
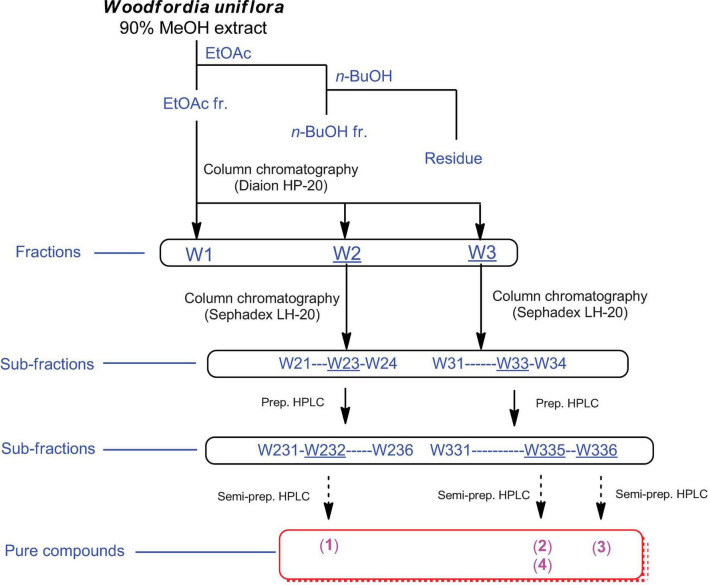
Separation scheme of compounds **1–4**.

**FIGURE 2:**
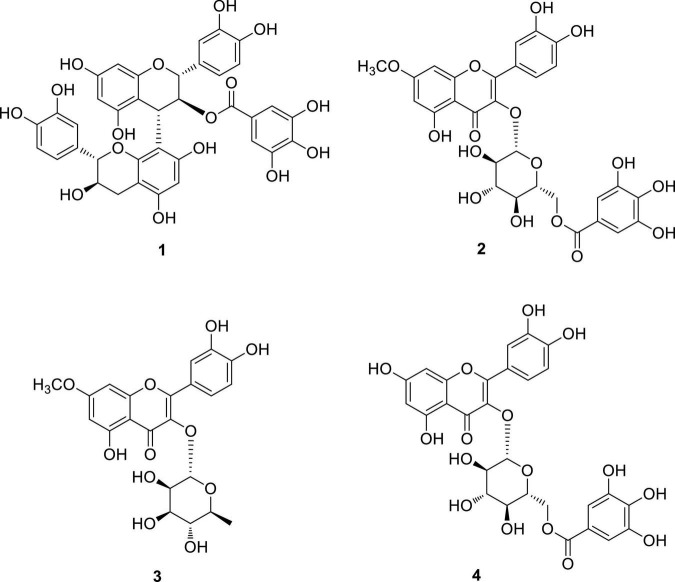
Chemical structures of compounds **1–4**.

Omani *W. uniflora* leaves were extracted with 90% aqueous MeOH to obtain the crude methanol extract after rotary evaporation. The crude extract was sequentially subjected to solvent partitioning using two organic solvents, EtOAc and *n*-BuOH, to yield each solvent fraction (Figure 1). LC/MS-based analysis of the solvent-partitioned fractions obtained in combination with our in-house UV library revealed that the EtOAc-soluble fraction had major peaks with characteristic features for interesting flavonoids. The intensive phytochemical investigation of the EtOAc-soluble fraction using successive column chromatography as well as preparative and semi-preparative HPLC purification monitored by LC/MS analysis led to the isolation of four flavonoid-type analogs (**1–4**) (Figure 1). The structures of compounds **1–4** (Figure 2) were determined to be procyanidin B3-3-*O*-gallate (**1**) (Saito et al., 2004), rhamnetin 3-*O*-(6″-galloyl)-β-D-glucopyranoside (**2**) (Yu et al., 2020b), rhamnetin 3-*O*-α-L-rhamnopyranoside (**3**) (Chung et al., 2004), and quercetin 3-*O*-(6″-galloyl)-β-D-glucopyranoside (**4**) (Kadota et al., 1990) by comparing their NMR spectral (Supplementary Figures 1–4) with ECD data with those previously reported in the literature, and the data from LC/MS analysis (Supplementary Figures 5–8).

### Compounds Synergize With Methicillin Against Methicillin-Resistant *Staphylococcus aureus* by Inhibiting Its Biofilm Formation

**FIGURE 3:**
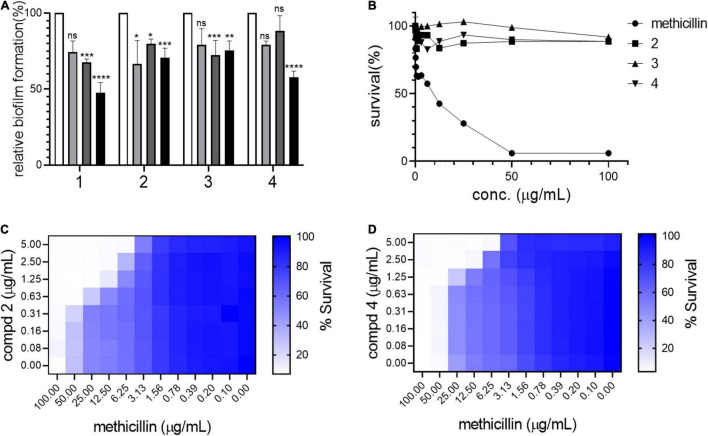
Compounds kill USA300 by resensitizing bacterial cells to methicillin. **(A)** Biofilm formation assays were performed at 0 (white bar), 5 (light gray), 20 (dark gray), and 50 (black) μg/mL of compounds **1–4**. Statistically significant differences were defined as *P ≤ 0.05; **P ≤ 0.01; ***P ≤ 0.001; and ****P ≤ 0.0001. **(B)** Survival of USA300 were measured under compound treatments. Compounds and methicillin combination treatment shows synergistic activity against USA300 **(C,D)**. Heatmap plots were generated from checkerboard assay for combination treatment of methicillin and compounds **2** and **4**.

Biofilm formation in *S. aureus* is an important remodeling of the bacterial cell envelope that contributes to the development of resistance against cell-wall targeting antibiotics including β-lactams (Schilcher and Horswill, 2020). The isolated compounds **1–4** were tested by treating bacterial cultures to determine their activity against biofilm formation by *S. aureus* USA300. As shown in Figure 3A, all compounds strongly inhibited biofilm formation in a dose-dependent manner (5–50 μg/mL). This result led us to test whether these compounds repress the *agrA* gene, a known central regulator of the biofilm formation pathway in *S. aureus* (Koenig et al., 2004). However, no significant reduction in the expression of *agrA* was found upon treatment with compounds in either shorter (1 h) or longer treatment (4 h), suggesting that the compounds inhibited the biofilm of *S. aureus* USA300 in the *agrA-independent* pathway (Supplementary Figure 9). Furthermore, as shown in Figure 3B, the antimicrobial effects of compounds **1–3** alone appeared to be marginal, although growth inhibition at their higher concentrations was observed. Our growth profiling experiment also demonstrated no significant reduction in specific growth rate of *S. aureus* USA300 at low concentrations of compounds **2** and **4**, while the specific growth rate was significantly reduced at higher concentrations (> 5 mg/mL) (Supplementary Figure 10).

This result suggested that our compounds might act by repressing other pathways related to biofilm formation in *S. aureus*. Since compounds **1–4** inhibited biofilm formation in *S. aureus* USA300, the potential synergistic effect with methicillin was examined using a checkerboard assay in which both methicillin and each compound were co-treated at serially diluted concentrations in combinations. Based on structural priority, compounds **2**, **3**, and **4** were selected for further analysis. As shown in Figures 3C,D, compounds **2** and **4** showed a strong synergetic effect while compound **3** showed only a marginal effect in the presence of methicillin (Supplementary Figure 11A). As shown in Supplementary Figures 11B,C, when compounds **2** and **4** were tested on another MRSA strain, MW2, again only compound **4** demonstrated synergistic effect with methicillin. Furthermore, when we tested compounds **2** and **4** against HG003, a methicillin sensitive *S. aureus* (MSSA) strain, none of the compounds showed synergistic effect (Supplementary Figures 11D,E). We thought that such differential response to the compounds results from unique MRSA cell envelope properties. Therefore, these results suggest that targeting MRSA-specific biofilm formation pathway can be an effective antibiotic resensitizing strategy against MRSA (Davies, 2003).

### Compounds **2** and **4** Lead to Abnormal Cell Morphology in *S. aureus* USA300

**FIGURE 4:**
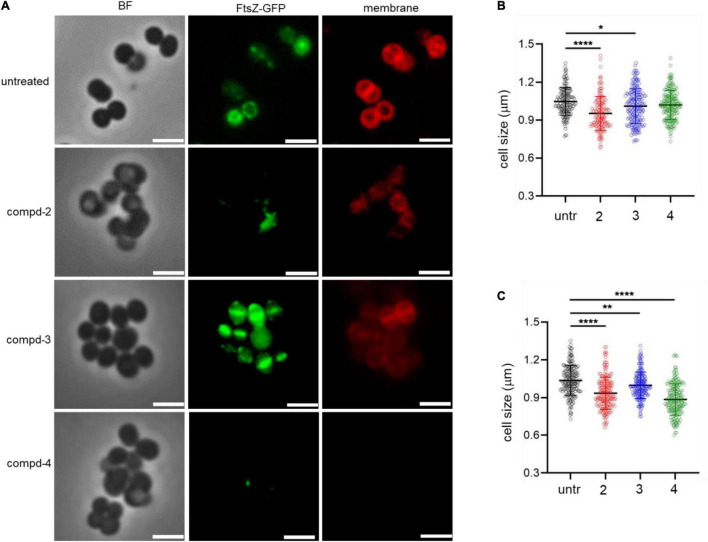
Compounds **2** and **4** inhibit the bacterial septal formation. **(A)** USA300/pLow-ftsZ-GFP were treated with 20 μg/mL of compounds and incubated at 37°C for 1 h. The cell membrane was stained using FM™ 4-64 Dye for 10 min. **(B,C)** Quantitative analysis of the cell size was performed at 5 μg/mL **(B)** and 20 μg/mL **(C)**. Scale bar = 2 μm. Microscopy images are representative of at least three experiments. Statistically significant differences were defined as *P ≤ 0.05; **P ≤ 0.01; and ****P ≤ 0.0001.

To test the terminal phenotypes of *S. aureus* USA300 in the presence of the compounds, bacterial division septa were analyzed by monitoring ectopically expressed GFP-tagged FtsZ protein that builds the septal ring in bacterial cells. To exclude the possible effects of methicillin on the cell envelope and division septa of *S. aureus* USA300, we treated the cells with the compounds alone. As shown in Figure 4, upon treatment with compounds **2** and **4**, signals from FtsZ-GFP were diffused and quenched significantly, indicating that the cells were not actively forming the Z-ring, unlike the untreated control, while compound **3** had only a minimal effect on the formation of division septa (Figure 4A). This result suggested that compounds **2** and **4** actively inhibited bacterial division although this mechanism of action repressed only or primarily biofilm formation because we found only negligible growth inhibition under these compounds alone (Figure 3B). We also performed cell size analysis by staining the cell membrane of *S. aureus* USA300. As shown in Figure 4A, the size of bacterial cells was found to be reduced upon treatment with compounds **2** or **4** (Figures 4B,C). In addition, we observed significant signal quenching from membrane staining. These results suggested that compounds **2** and **4** could generate abnormalities in the cellular metabolism of *S. aureus* USA300, causing a reduction in biofilm formation. These metabolisms might not be directly related to the *agrA* pathways but may involve other carbon metabolisms including central carbon and amino acid metabolism that provide precursors for biofilm biosynthesis in *S. aureus* USA300 (Davies, 2003; Verderosa et al., 2019).

### Compounds **2** and **4** Affect Metabolic Remodeling in *S. aureus* USA300

**FIGURE 5:**
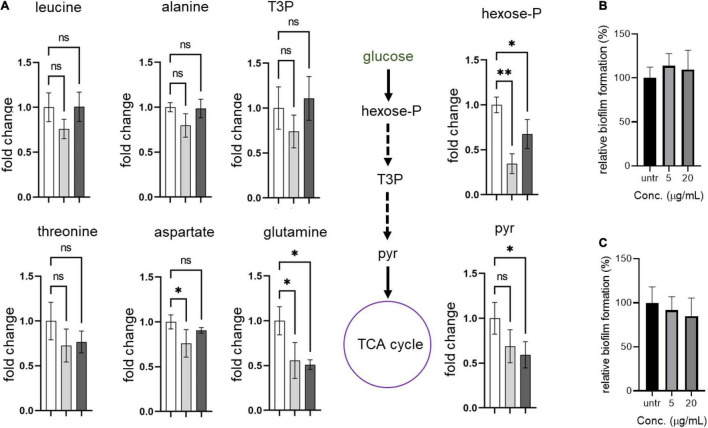
Compounds lead to metabolic remodeling in USA 300. **(A)** USA300 were treated with 5 μg/mL of compound **2** or **4** for 1 h. Fold change is expressed relative to the untreated culture with three biological replicates (*n* = 3) ± standard deviation. Statistically significant differences were defined as *P ≤ 0.05 and ^**^P ≤ 0.01. Open bar represents untreated group, while gray and black bars represent groups treated with compounds **2** and **4**, respectively. **(B,C)** Glutamine antagonizes the anti-biofilm activity of compounds **2** and **4**. Quantification of relative biofilm formation of *S. aureus* USA300 was performed in the presence of 1 mg/mL glutamine and compound **2 (B)** or **4 (C)**.Hexose-P, glucose-6-phosphate and its isomers; T3P, triose 3-phosphates (dihydroxyacetone phosphate and glyceraldehyde phosphate); pyr, pyruvate.

Next, we hypothesized that the formation of abnormal cells in *S. aureus* USA300 due to compounds **2** and **4** could be caused by changes in central carbon and amino acid metabolisms. To test this possibility, we chose key metabolic nodes in glycolysis and amino acid biosynthesis and performed intracellular metabolite analysis in *S. aureus* USA300 cells in the presence of either **2** or **4**. As shown in Figure 5, the levels of hexose-phosphate and pyruvate were significantly reduced upon treatment with the compounds, while we found that the level of T3P was changed only marginally. The reduction of these two key metabolites in glycolysis can lead to lower production of energy, causing slower growth and formation of smaller cells. Importantly, the reduction in hexose-phosphate can result in lower flow in the production of glycolipids, thereby inhibiting biofilm formation in *S. aureus* because hexose precursors of glycolipids are essential constituents of biofilms in bacterial cells (Schilcher and Horswill, 2020). Among the amino acids analyzed, we found that the level of glutamine was significantly reduced in the presence of either **2** or **4**. In *S. aureus*, glutamine is primarily synthesized by glutamine synthetase; therefore, this enzyme has been implicated as a target of anti-biofilm activity (Cui et al., 2019; Sachla and Helmann, 2021). However, we found that other amino acids such as leucine, alanine, threonine, and aspartate remained unchanged. To further test whether glutamine antagonizes the effects of compounds **2** and **4**, we treated *S. aureus* USA300 culture with the compounds in the presence of glutamine; and interestingly we found that supplementation with glutamine significantly antagonized the effects of compounds **2** and **4** (Figures 5B,C), which validated our metabolite analysis results. These results of metabolite analysis suggest that our compounds might not work through *agrA* signaling (Supplementary Figure 9) but rather modulate carbon or glutamine/glutamate metabolism, leading to reduction of precursors of biofilm formation. Furthermore, this metabolic remodeling of amino acid metabolism can result in an imbalance in the pathways for precursors required for the stem peptide found in the peptidoglycan of bacterial cells, an observation consistent with our microscopic analysis (Figure 4).

## Conclusion

In conclusion, phytochemical analysis of bioactive natural products found in the methanolic extract of *W. uniflora* leaves led to the isolation and identification of four flavonoid-type analogs procyanidin B3-3-*O*-gallate (**1**), rhamnetin 3-*O*-(6″-galloyl)-β-D-glucopyranoside (**2**), rhamnetin 3-*O*-α-L-rhamnopyranoside (**3**), and quercetin 3-*O*-(6″-galloyl)-β-D-glucopyranoside (**4**). We found that treatment of *S. aureus* (strain USA300) with compounds **2** or **4** resulted in a significant reduction in the MRSA biofilm formation. Although these compounds alone have only marginal antibacterial potency, they have shown a strong synergistic potency with methicillin against *S. aureus*. Our metabolite analysis and microscopy suggest that this synergistic effect can be achieved by metabolic remodeling in glycolysis and glutamine synthesis that causes an imbalance in the flow of precursors for biosynthesis of both bacterial cell envelopes and biofilm. Our results underscore those of many previous studies that showed an interplay between biofilm formation and antibiotic resistance, and that biofilm formation in bacterial cells can be an outstanding drug target. Lastly, we note that compounds **2** and **4** showed no significant cytotoxicity (Supplementary Figure 12), therefore, natural product compounds identified in our study are potent against biofilm formation and offer an alternative therapeutic approach by synergizing with clinically used antibiotics to re-sensitize important drug-resistant pathogenic bacteria, including MRSA, to antibiotics.

## Data Availability Statement

The original contributions presented in the study are included in the article/Supplementary Material, further inquiries can be directed to the corresponding author/s.

## Author Contributions

WL and KK conceived the project. JY, J-HK, LR, and IK performed the experiments. LR contributed to providing resources. JY, WL, and KK wrote the manuscript with help from the other authors. All authors contributed to the article and approved the submitted version.

## Conflict of Interest

The authors declare that the research was conducted in the absence of any commercial or financial relationships that could be construed as a potential conflict of interest.

## Publisher’s Note

All claims expressed in this article are solely those of the authors and do not necessarily represent those of their affiliated organizations, or those of the publisher, the editors and the reviewers. Any product that may be evaluated in this article, or claim that may be made by its manufacturer, is not guaranteed or endorsed by the publisher.
