# Supplementary material for: Potential Antimicrobial Activity of Galloyl-Flavonoid Glycosides From Woodfordia uniflora Against Methicillin-Resistant Staphylococcus aureus
Source: Front Microbiol. 2021 Nov 26;12:784504. doi: 10.3389/fmicb.2021.784504 (PMC8662356; doi:10.3389/fmicb.2021.784504)
Supplement: Supplementary file 1 [file Data_Sheet_1.PDF]

Supplementary Material

Potential Antimicrobial Activity of Galloyl-flavonoid Glycosides from *Woodfordia uniflora* against Methicillin-resistant *Staphylococcus aureus*

Jae Sik Yu[1,a], Ji-Hoon Kim[1,a], Luay Rashan[2], Inseo Kim[1], Wonsik Lee[1]*, and Ki Hyun Kim[1]*

[1]School of Pharmacy, Sungkyunkwan University, Suwon, 16419, Republic of Korea

[2]Research Center, Biodiversity Unit, Dhofar University, Salalah, Sultanate of Oman

[a]These authors contributed equally to this work

*** Correspondence:**

Ki Hyun Kim - khkim83@skku.edu

Wonsik Lee - wonsik.lee@skku.edu

Contents

Figures S1-S4. NMR spectroscopic data of compounds 1-4

Figures S5-S9 LC/MS data of compounds 1-4

Figure S9. *agrA*-independent anti-biofilm activity of compounds.

Figure S10. Compounds 2 and 4 have growth inhibitory activity at high concentrations.

Figure S11. Compounds 2 and 4 have synergetic effects only in MRSA.

Figure S12. Cytotoxicity of compounds 2 and 4.

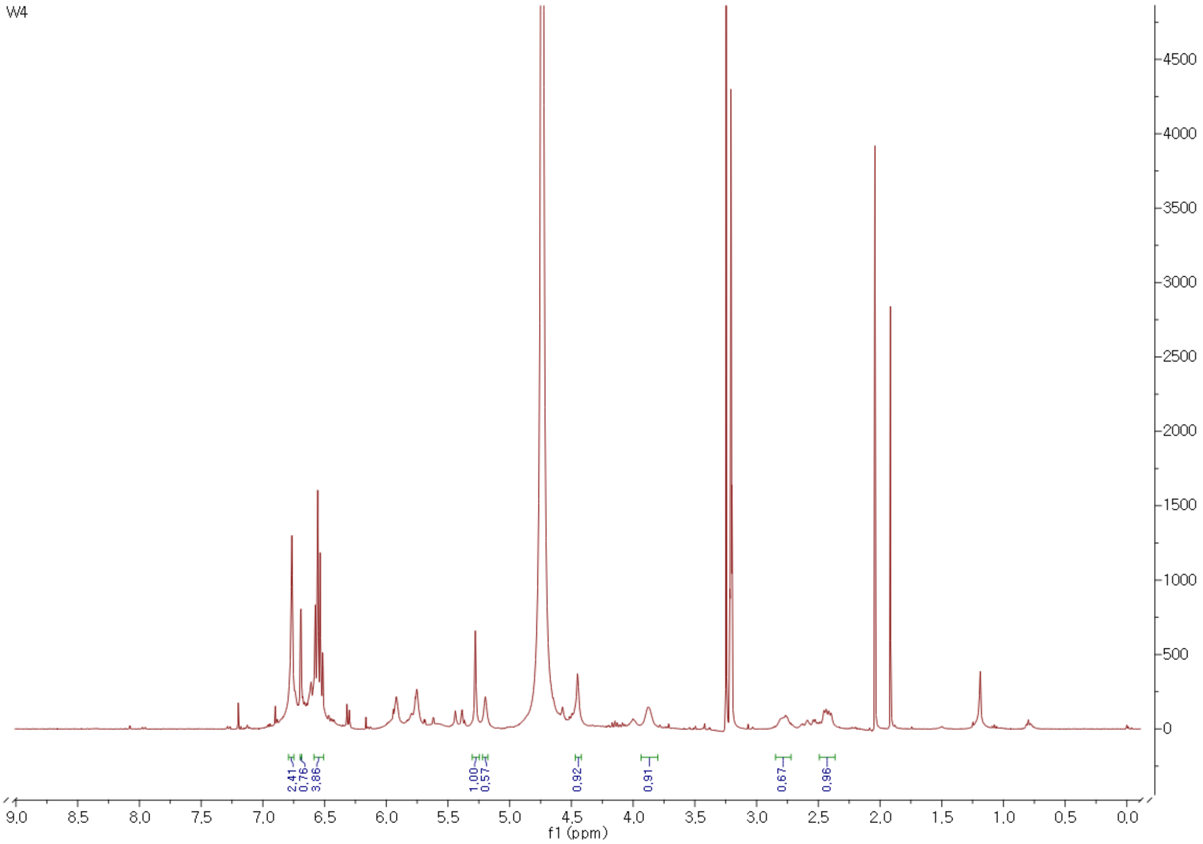

Figure S1. ^{1}H NMR spectrum of **1** (CD_3OD, 850 MHz)

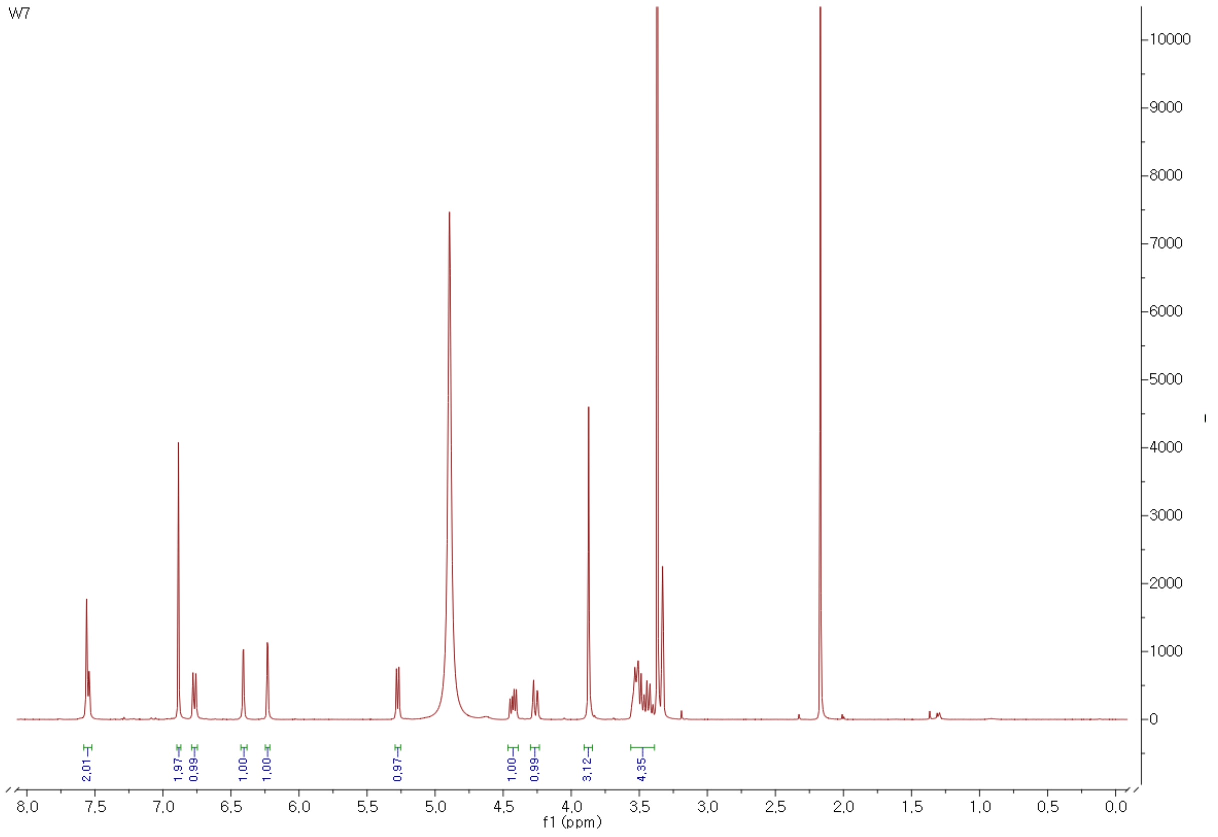

Figure S2. ^{1}H NMR spectrum of **2** (CD_3OD, 850 MHz)

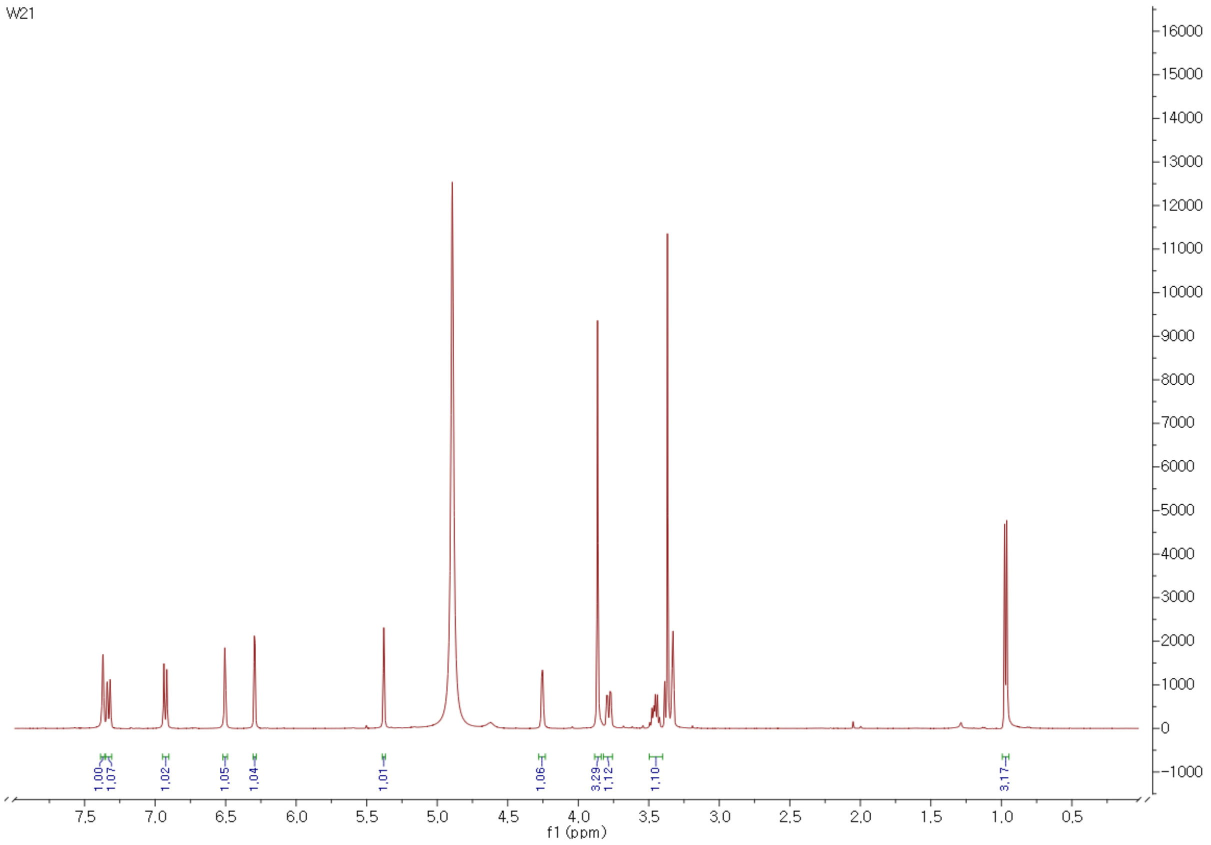

Figure S3. ^{1}H NMR spectrum of **3** (CD_3OD, 850 MHz)

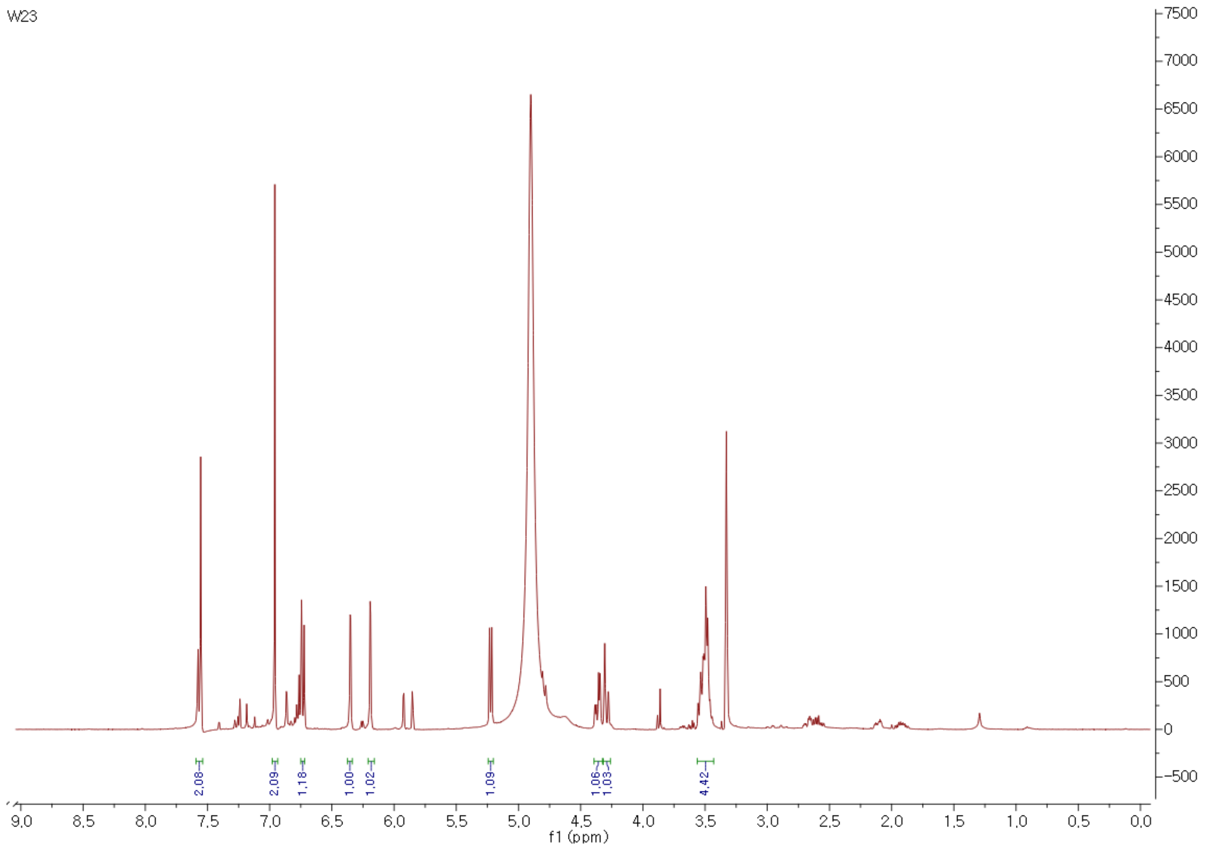

Figure S4. ^{1}H NMR spectrum of **4** (CD_3OD, 850 MHz)

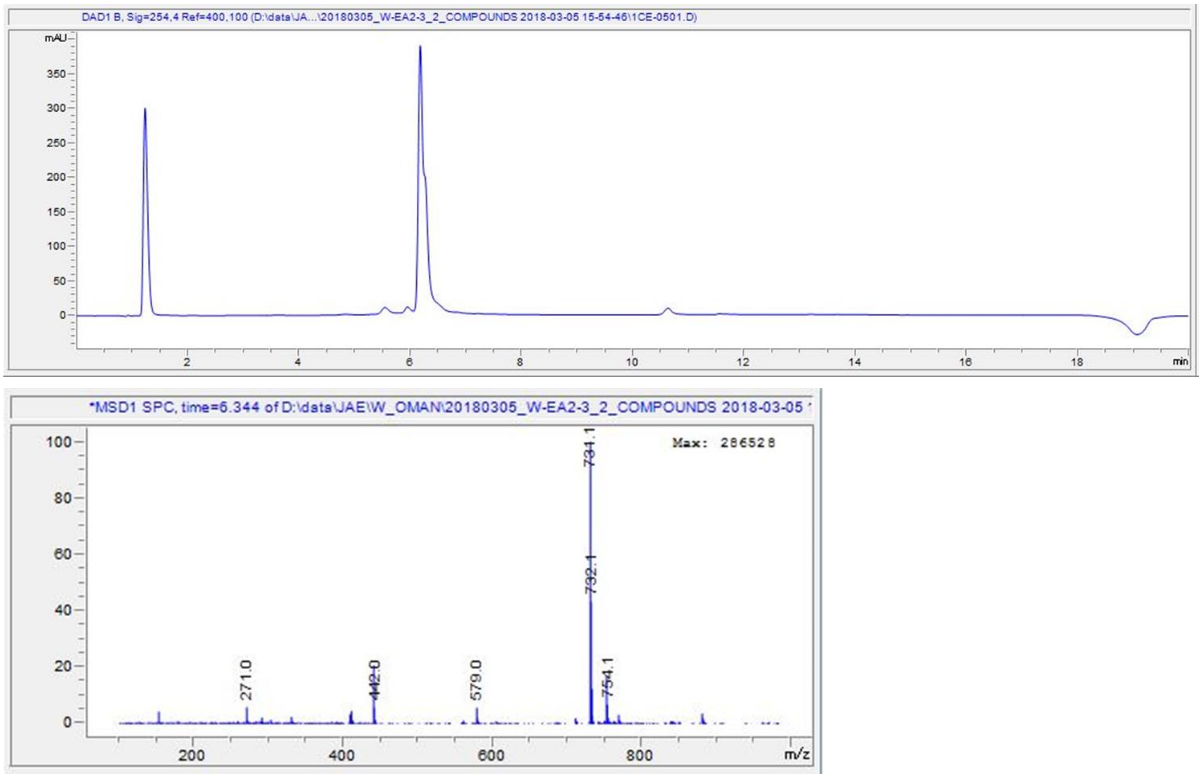

Figure S5. LC/MS data of **1** (detection wavelength was set at 254 nm, and ESI-MS data of **1** at *m/z* 731.1 $[M+H]^+$ in the positive mode)

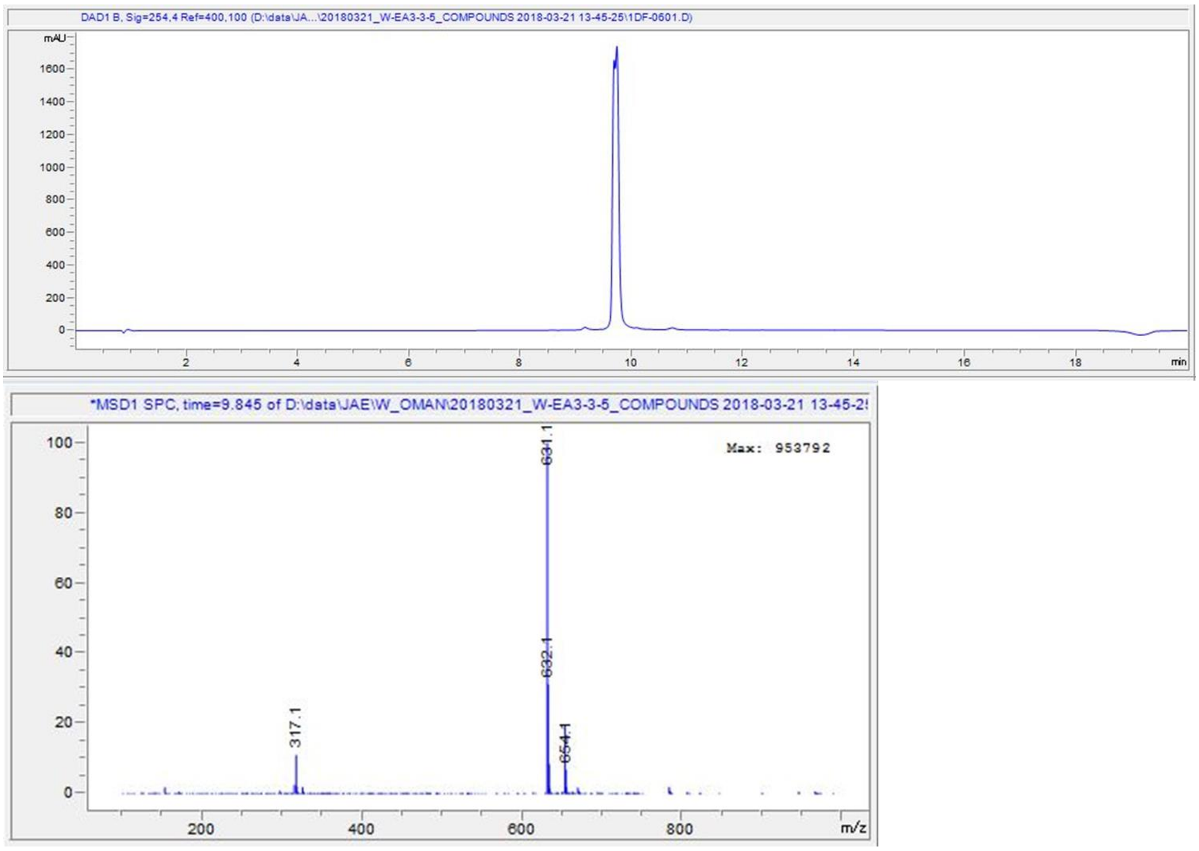

Figure S6. LC/MS data of **2** (detection wavelength was set at 254 nm, and ESI-MS data of **2** at *m/z* 631.1 $[M+H]^+$ in the positive mode)

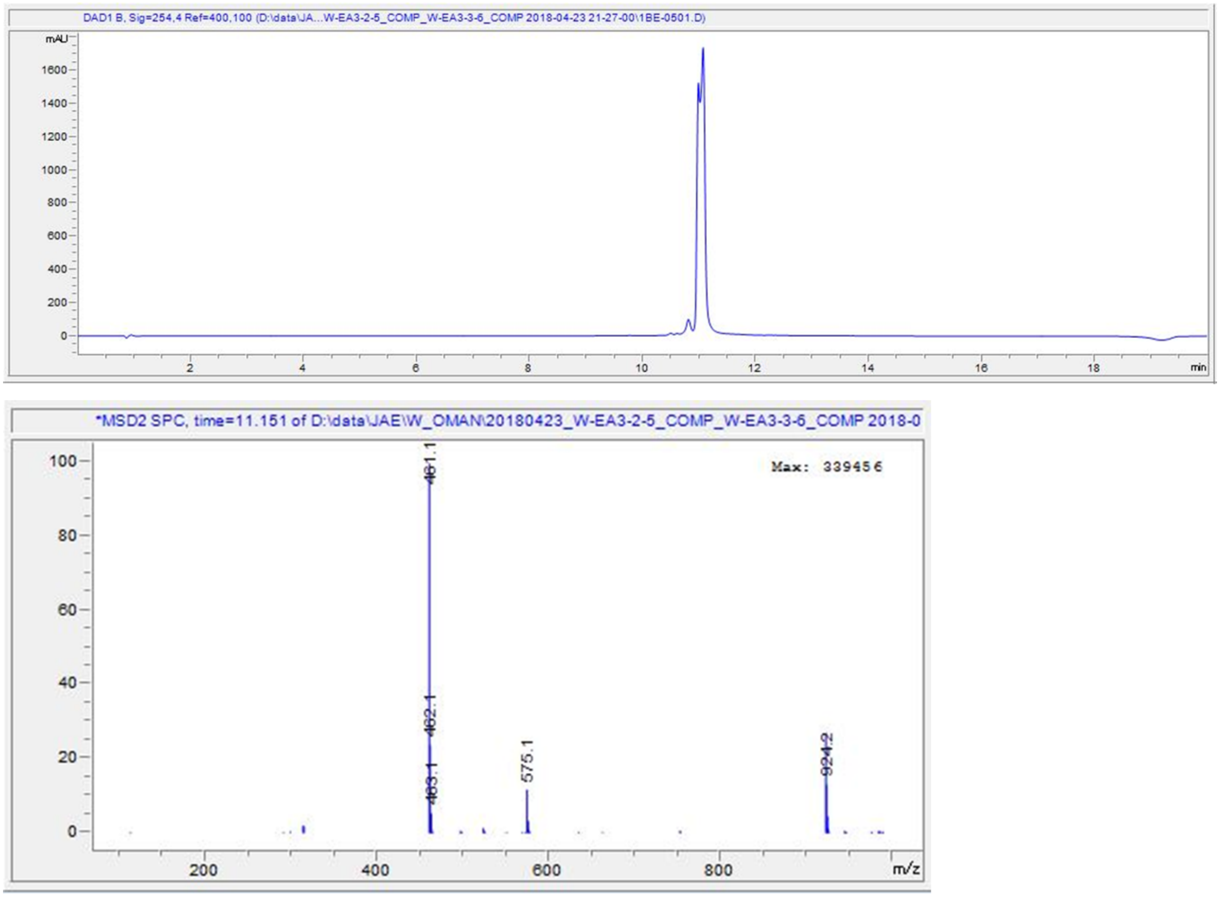

Figure S7. LC/MS data of **3** (detection wavelength was set at 254 nm, and ESI-MS data of **3** at *m/z* 461.1 $[M-H]^-$ in the negative mode)

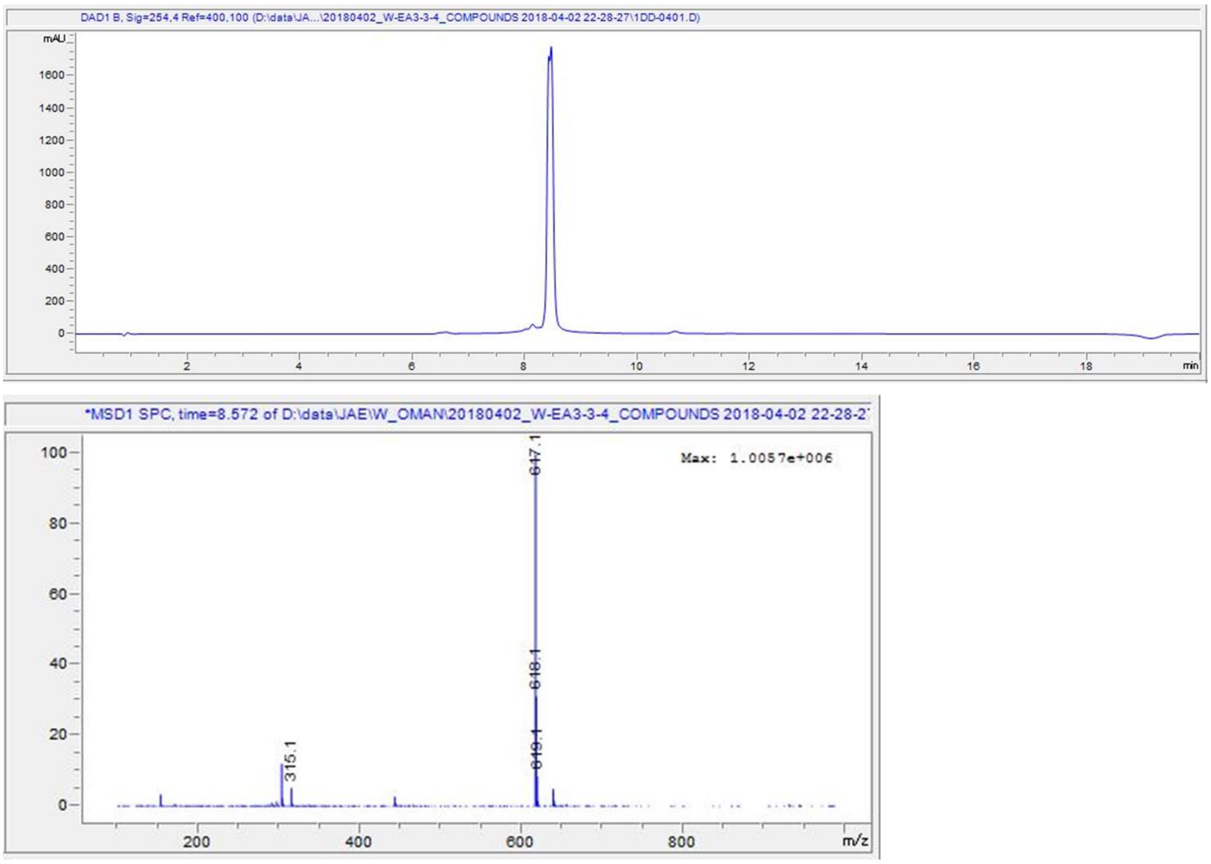

Figure S8. LC/MS data of **4** (detection wavelength was set at 254 nm, and ESI-MS data of **4** at *m/z* 617.1 $[M+H]^+$ in the positive mode)

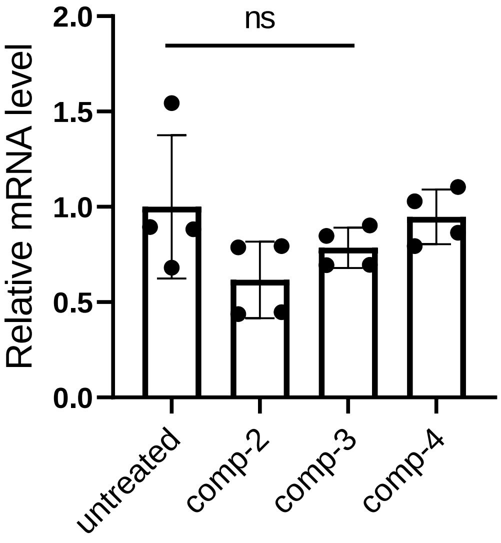

Figure S9. *agrA*-independent anti-biofilm activity of compounds. Quantitative real-time PCR (qPCR) was performed to assess the expression of *agrA* of USA300 cultures that were incubated with compounds at a concentration of 5 μg/mL. Error bars indicate standard deviation (SD).

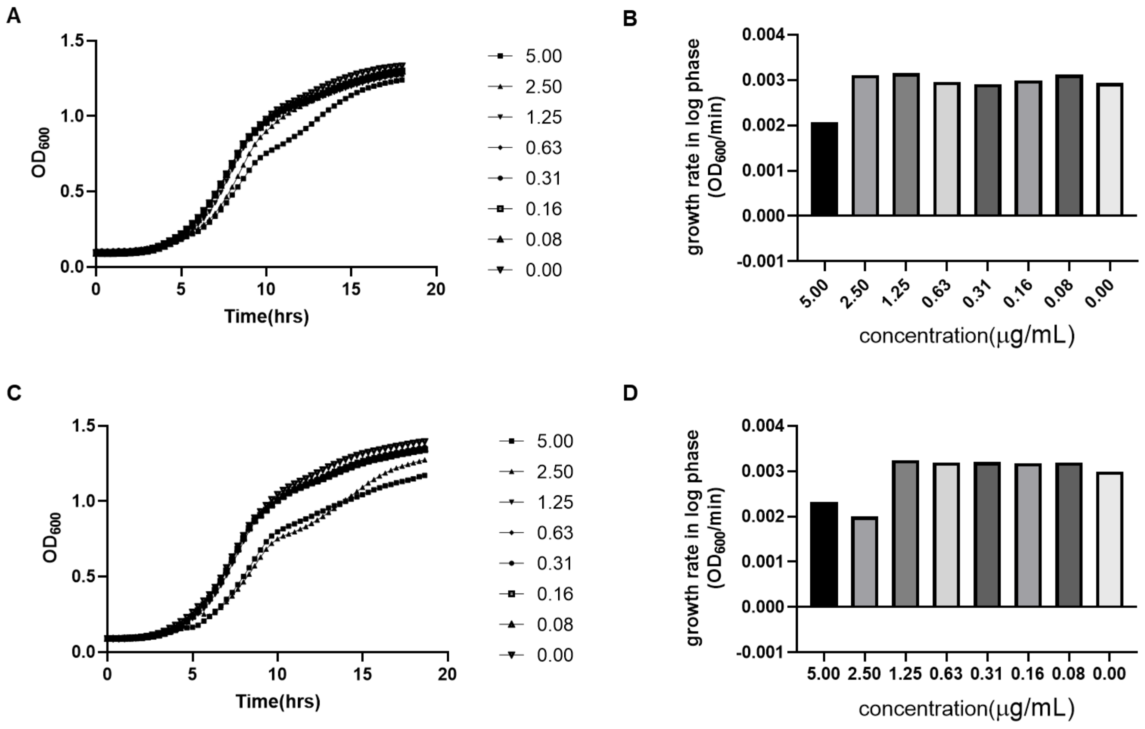

Figure S10. Compounds 2 and 4 have growth inhibitory activity at high concentrations. Growth rate profiling was performed on USA300 treated with various concentrations of the compounds **2** (A) and **4** (C) and represented as a growth curve. Growth rates at log phase for compounds **2** (B) and **4** (D) was calculated using growth curve data.

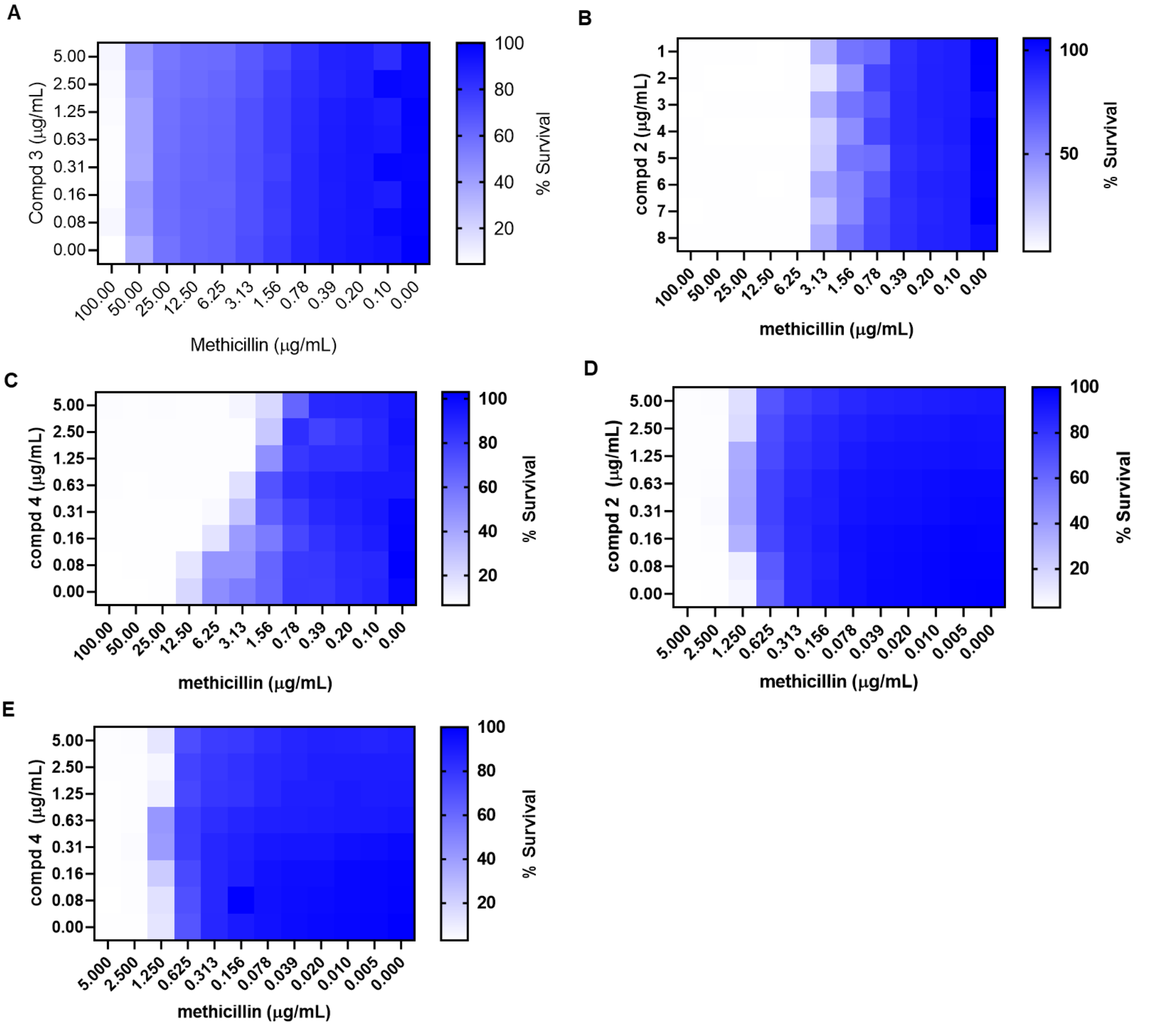

Figure S11. Compounds 2 and 4 have synergetic effects only in MRSA. Heatmap plots were created using data from checkerboard assay for combination treatment of methicillin and compound **3** for *S. aureus* USA300 (A), methicillin and compounds **2** and **4** for *S. aureus* MW2 (B and C), and for *S. aureus* HG003 (D and E).

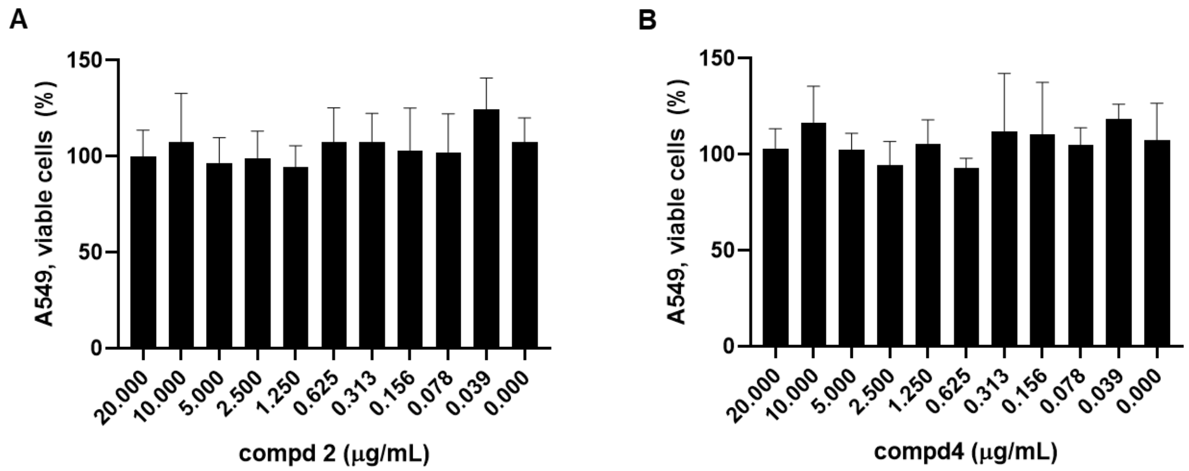

Figure S12. Cytotoxicity of compounds 2 and 4. Cytotoxicity of compounds **2** (A) and **4** (B) were examined using A549 cell line treated with the compounds at given concentrations for 16 hours.
